# Advances in the study of RNA-binding proteins in diabetic complications

**DOI:** 10.1016/j.molmet.2022.101515

**Published:** 2022-05-18

**Authors:** Xinyue Chen, Jiaqiang Wu, Zhangwang Li, Jiashu Han, Panpan Xia, Yunfeng Shen, Jianyong Ma, Xiao Liu, Jing Zhang, Peng Yu

**Affiliations:** 1 The Second Clinical Medical College of Nanchang University, the Second Affiliated Hospital of Nanchang University, Nanchang, China; 2 Department of Thoracic Surgery, Peking Union Medical College Hospital, Beijing 100730, China; 3 Department of Anesthesiology, The Second Affiliated Hospital of Nanchang University, Jiangxi, Nanchang 330006, China; 4 Department of Metabolism and Endocrinology, the Second Affiliated Hospital of Nanchang University, Nanchang, China; 5 Department of Pharmacology and Systems Physiology, University of Cincinnati College of Medicine, USA; 6 Department of Cardiology, Sun Yat-sen Memorial Hospital, Sun Yat-sen University, Guangzhou, China

**Keywords:** Diabetes, Diabetic complications, RNA binding proteins, Therapy, AREs, AU element-rich, ATG5, Autophagy-related gene 5, CSD, Cold shock domain, CUGBP1, CUG-binding protein 1, EMT, Epithelial mesenchymal transition, EIF4E, Eukaryotic translation initiation factor 4E, FoxO1, Forkhead box protein O1, FTO, Fat mass and obesity-associated protein, GLUT4, Glucose transporter 4, GSIS, Glucose-stimulated insulin secretion, HuR, Human antigen R, hnRNP, Heterogeneous ribonucleoprotein, IR, Insulin resistance, INSIG1, Insulin-induced gene 1, IGF2, Insulin-like growth factor 2, IMP2, IGF2 mRNA-binding protein 2, KH, K homology, Lamb2, Laminin-β 2, MC, Mesangial cell, Mfn2, Mitofusin-2, MBNL1, Muscleblind-like 1, RBPs, RNA binding proteins, SRF, Serum response factor, TTP, Tristetraprolin, ZBP, Z-DNA-binding protein

## Abstract

**Background:**

It has been reported that diabetes mellitus affects 435 million people globally as a primary health care problem. Despite many therapies available, many diabetes remains uncontrolled, giving rise to irreversible diabetic complications that pose significant risks to patients’ wellbeing and survival.

**Scope of Review:**

In recent years, as much effort is put into elucidating the posttranscriptional gene regulation network of diabetes and diabetic complications; RNA binding proteins (RBPs) are found to be vital. RBPs regulate gene expression through various post-transcriptional mechanisms, including alternative splicing, RNA export, messenger RNA translation, RNA degradation, and RNA stabilization.

**Major Conclusions:**

Here, we summarized recent studies on the roles and mechanisms of RBPs in mediating abnormal gene expression in diabetes and its complications. Moreover, we discussed the potential and theoretical basis of RBPs to treat diabetes and its complications.

## Highlights

• Mechanisms of action of RBPs involved in diabetic complications are summarized and elucidated.
• We discuss the theoretical basis and potential of RBPs for the treatment of diabetes and its complications.
• We summarize the possible effective drugs for diabetes based on RBPs promoting the development of future therapeutic drugs.

## 1 Introduction

The incidence of diabetes mellitus (DM) and its complications pose a major threat to global health. According to a survey by the International Diabetes Federation (IDF), DM is a major health problem affecting 435 million people worldwide [1]. In 2021, 537 million adults between the ages of 20–79 worldwide (representing 10.5% of all adults in this age group) had diabetes [2]. The prevalence of diabetes is constantly increasing, as is its impact on the lives and health of those living with it. Current data shows that diabetes is the ninth major reason for death [3]. Apart from the disease itself, the complications of diabetes also threaten public health. In a cross-sectional study of 1,542 people with type 2 diabetes, over 50% had at least one chronic diabetic complication [4]. Cardiovascular and cerebrovascular complications are the leading cause of death in people with diabetes mellitus [5], with a high prevalence of 30.1% and 6.8% respectively [4]. Other complications of diabetes such as retinal and peripheral neuropathy also have a serious effect on the patient's life quality, causing huge health and economic burdens on society [6].

The pathogenesis of diabetes and its complications is a sophisticated process that involves many different pathways and multi-layered regulation. Some classical mechanisms of diabetes, such as insulin resistance (IR), inflammation, and oxidative stress are well studied [7]. Skeletal muscle IR is an important feature of type 1 diabetes (T1D). Impaired upregulation of insulin-stimulated GLUT4 (glucose transporter-4) messenger RNA (mRNA) leads to reduced glucose transport into myocytes [[8], [9], [10]]. Low level of inflammation and immune activation are considered to be important in the development of type 2 diabetes (T2D) [11]. In particular, interleukin-1β-mediated activation of the inflammasome of NLR family pyrin domain containing 3 (NLRP3) is strongly linked to the development of T2D [12]. In addition, oxidative stress contributes to the pathogenesis of diabetes mellitus and its complications. Oxidative stress occurs when radicals are formed at a rate greater than their metabolic rate. and the excessive accumulation of radicals leads to the toxic effects [13]. Reactive oxygen species (ROS) such as hydrogen peroxide (H_2_O_2_) are important factors in pancreatic β-cell, functioning in cellular signaling processes and regulation of glucose-stimulated insulin secretion (GSIS) [14,15]. As all these above-mentioned mechanisms are downstream effectors, more researchers are looking for the common 'keys' to these mechanisms, namely their upstream regulators, that acts as a master regulatory factor to the development and progression of DM and its complications. Recent studies have then found that RNA binding proteins (RBPs) such as human antigen R (HuR) and TTP (Tristetraprolin) are directly involved in vasculopathy in diabetes or diabetic complications by regulating the expression and stability of vascular endothelial growth factor (VEGF) mRNA [[16], [17], [18]], while LIN28 mediates the handling and ripening of let7 microRNA (miRNA), which promotes the uptake of blood glucose by tissue cells to inhibit the progression of DM and its complications [[19], [20], [21]].

RBPs are essential post-transcriptional controllers of RNA expression. RBP-RNA interactions compose a complex network, and dysfunction in the network is at the root of many diseases [22]. RBP is a large family protein, including over 2000 members that each interacts with their specific transcripts during various RNA-driven processes. RBPs have diverse functions in RNA export, alternative splicing, mRNA translation, RNA stabilization, and RNA degradation [[23], [24], [25], [26]]. Current studies have shown that RBP is involved many diseases, particularly metabolic diseases such as hyperuricemia, hyperlipidemia, hypertension, non-alcoholic fatty liver disease (NAFLD), and diabetes [27,28]. As an example, RBP fox-1 homolog 2 (RBFOX-2) is involved in coronary heart disease by binding to the (U) GCAUG pattern in RNA to regulate alternative splicing [29]. RBP Quaking (QKI) is involved in the treatment of NAFLD by modulating the PPARα expression, activating the transcription factor forkhead box protein O1 (FoxO1), and inhibiting triacylglycerol synthesis [30]. RBP, as an upstream factor, has also become a key tool and target. For example, CYC27 exerts antidiabetic functions by sensitizing insulin signaling channels and regulating RNA splicing-associated RBPs [31].

There is growing interest in the role of RBPs in diabetes and diabetic complications. Recent human genetic studies suggest that polymorphisms in RBPs are closely associated with diabetes [32]. Chao et al. identified hnRNP F, an RBP participating in the diabetic kidney disease (DKD) development through regulation of Angiotensinogen (Agt) and transforming growth factor-β1 (TGF-β1) gene expression (discussed below) [33]. These findings and studies suggest that our RBP abnormalities are connected to the development of DM and associated complications, and that elucidating the above mechanisms is important to provide new therapeutic targets and strategies. This paper reviews the mechanisms of the contribution of different RBPs in the development of diabetes and its complications.

## 2 RBPs

### 2.1 Biogenesis

RBPs are multi-faceted players in transcriptional regulation, taking part in the entire lifecycle of RNA [34]. RBPs can recognize and interact with RNA-binding domains (RBDs), thus forming ribonucleoproteins (RNPs) for the regulation of RNA stability, alternative pre-mRNA splicing, mRNA decay, translocation, post-translational nucleotide modifications, and RNA localization [35]. RNA recognition motifs (RRMs) are the most common and well-studied RBDs [36]. Zinc finger family also contributes to RRMs. It has been reported that zinc finger family allows transcription factor IIIA to discriminate DNA from RNA by leveraging electrostatic interactions with protein side chains that capture RNA loops [37]. In addition, K homology (KH) domain was first discovered in heterogeneous nuclear ribonucleoprotein K (hnRNP K) [38]. As with RRMs, many KH domains in RBPs can independently increase binding specificity. Pumilio homology domain, pentatricopeptide repeat, pseudouridine synthase and archaeosine transglycoslyase (PUA), and cold shock domain (CSD) also play critical roles in RNA-RBP interaction [39].

### 2.2 Functions

**Figure 1:**
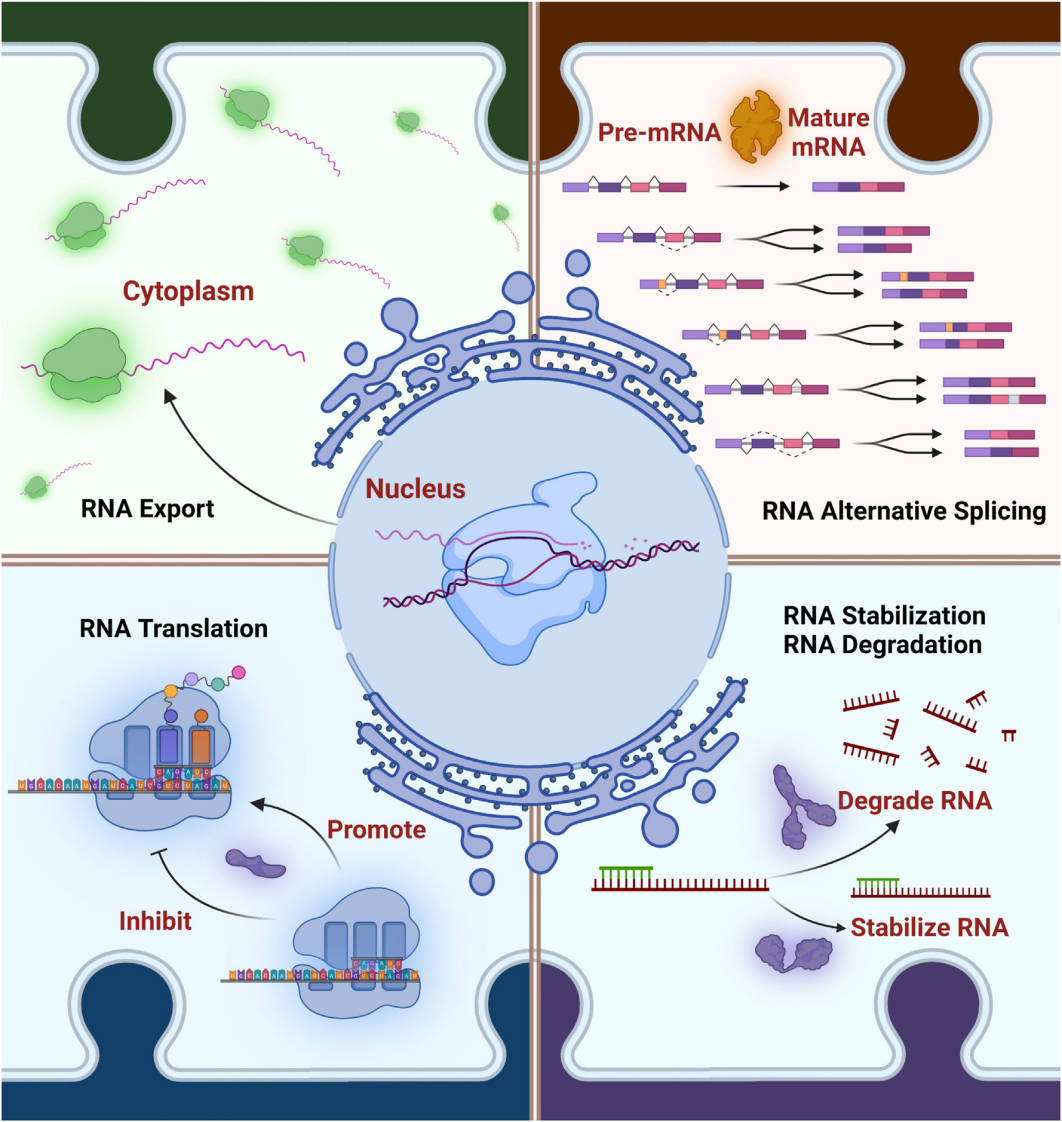
**Four main functions of RNA binding protein.** According to the current studies, there are four main functions of RNA binding proteins (RBPs): RNA export, RNA translation, alternative splicing, RNA stabilization and RNA degradation. The life path of a mRNA molecule from transcription to the generation of functional mature mRNA is an intricate process governed by many different RBPs. Alternative splicing is an important process for RNA transcription. As much as 95% of pre-mRNAs in mammalian cells undergo governed by a unique set of RBPs and their corresponding RNA binding sites. Additionally, RBPs can promote or inhibit RNA translation process by binding to the corresponding RNA. Also, RBPs can interact with deadenylating enzymes to remove the 3′ poly-A tail, or decapping enzymes to remove the 5′cap, thus inducing RNA degradation. Generally, RBPs can affect the function of RNA through the above four pathways.

There are four main functions of RBPs: alternative splicing, RNA export, mRNA translation, RNA degradation, and stabilization [[23], [24], [25], [26]] (Figure 1). The life path of a mRNA molecule from transcription to the generation of functional mature mRNA is an intricate process governed by many different RBPs, such as IGF2BP Family and Musashi protein family [40,41]. There are at least 1200 verified RBPs in the currently annotated human genome, as well as new one under investigation [25].

As much as 95% of pre-mRNAs in mammalian cells undergo alternative splicing governed by a unique set of RBPs and their corresponding RNA binding sites [42]. There are many relevant regulators, each with different functions under specific conditions [43]. A study found that Zinc finger Matrin-Type 3 makes oncogenic CD44 variants splicing be inhibited in colorectal carcinoma [44]. T1D is caused by autoimmune-mediated destruction of pancreatic β cells that produce insulin [45]. Alternative splicing plays a critical role in diabetes progression. It has been reported that alternative splicing impacts the β-islet cell function and autoimmunity [46]. Elavl4, RBFOX2, RBFOX1 and SRSF6 are important alternative splicing regulators in diabetic pancreatic β cells [[47], [48], [49], [50], [51]]. Polypyrimidine tract-binding protein 1 (PTBP1), CUGBP Elav-like family member (CELF) and RBFOX2 are associated with aberrant alternative splicing in diabetes [[52], [53], [54], [55]].

The rate-limiting initiation step is the most tightly regulated step of mRNA translation [56]. RBP eukaryotic translation initiation factor 4E (EIF4E) can participate in eukaryotic translation initiation by forming the eIF4F complex together with EIF-4A and EIF-4G. Thus, by binding to the 5′ end cap structure of mRNA, EIF-4E can bring eIF4A to the 5′ end of mRNA, allowing EIF-4A to exert its decapping activity to open the 5′ end secondary structure of mRNA [57,58]. Removal of the 5′ cap structure by decapping enzymes induces mRNA degradation [59]. RBPs can interact with deadenylating enzymes to remove the 3′ poly-A tail, or decapping enzymes to remove the 5′cap, thus inducing RNA degradation [23,60]. For example, RBP HuR targets the 3′- untranslated regions (UTR) region of IL-6 mRNA to enhances its stabilization in periodontitis [61].

### 2.3 Binding to or interacting with RNA

There is increasing evidence that RNA-RBPs interaction are crucial in various diseases that include diabetes, cardiovascular disease, cancer and neurodegenerative diseases [[62], [63], [64], [65], [66]]. Transactive response DNA binding protein 43 (TDP-43) is a highly conserved RBP, belonging to the heterogeneous ribonucleoprotein (hnRNP) family. TDP-43 is first found to inhibit TAT-induced HIV-1 transcription [67]. It has also been reported to regulate the transport and localization of specific mRNAs in dendrites and axons, and its regulatory function declines in neurodegenerative diseases [68]. A-kinase anchor protein 8 acts as an alternative splicing regulatory factor that impedes epithelial mesenchymal transition (EMT) and breast cancer metastasis [69]. RBP RBM38 could suppress the development of colorectal cancer by competing with miR-92a-3p for binding to PTEN 3′UTR [70]. It has been reported that RBP RBM24 expression is high in human and mouse heart. Global splicing profiling revealed that RBM24 modulates genes related to cardiovascular diseases. A study found that inhibition of RBM24 induced dysregulation of alternative splicing, thus resulting in the occurrence of cardiomyopathy [71]. In recent years, there is growing interest in the function of RBPs in diabetes and its complications. Recent human genetic studies suggest that polymorphism in RBPs is closely associated with diabetes [32]. In this review, we will review the role of RBPs in diabetes and relevant complications, and we will discuss potential therapeutical strategy.

## 3 RBPs implicated in the development of multiple diabetic complications

### 3.1 Microangiopathy

Microangiopathy is clinical manifestations of morphological, structural and functional changes of the microvasculature. Diabetic nephropathy and diabetic retinopathy are the main diabetic microvascular complications [72]. The most prevalent mechanisms leading to endothelial dysfunction are oxidative stress and enhanced levels of ROS. Endothelial cells and their main production, nitric oxide and prostacyclin, have a crucial role in the regulation of vascular homeostasis. Vascular endothelial cell dysfunction has been identified as a primary mediator of diabetic vascular complications. Endothelial dysfunction caused by diabetes is a key and initiating factor in the development of diabetic vascular complications [73,74]. Non-coding RNAs have a particularly functional role in microvascular dysfunction. MiRNAs, non-coding RNAs, and associated RBPs are implicated in many cores cellular processes [75,76]. For example, Yan et al. revealed a modulatory effect of lncRNA myocardial infarction-associated transcripts in diabetes-induced microvascular dysfunction [77]. HuR, hnRNP K, hnRNP F, and LIN28 are mal-regulated in diabetic nephropathy [78]. The significant role of RBPs in diabetic microangiopathy has been demonstrated by numerous studies demonstrating possible regulatory mechanisms.

Translation-regulated RNA-binding proteins (TTR-RBPs) are important proteins that play a role in modulating gene expression patterns. TTR-RBPs control the expression of genes at the post-transcriptional level by cooperating or competing with specific miRNAs [79], affecting processes such as pre-mRNA splicing, mRNA transfer to the cytoplasm, turnover, storage and translation [80]. Although there are a few TTR-RBPs that only regulate specific post-transcriptional processes such as Tristetraprolin (TTP) and KH-type splicing regulatory protein-mediated mRNA splicing [81,82], the vast majority of TTR-RBPs are able to regulate a wide range of post-transcriptional processes. As an example, HuR and nuclear factor 90 regulate both mRNA stability and translation [83]. We will review three specific RBPs in detail to illustrate the important role of RBPs in diabetic microangiopathy.

#### 3.1.1 Human antigen R

Human antigen R (HuR) is among the most well-studied TTR-RBPs [84]. HuR stabilizes many of mRNAs and/or modulates their translation [85]. Many mechanisms elevate the level of HuR in diabetic states. According to Amadio et al., STZ-induced diabetic rats had a 62% increase in HuR protein levels compared to controls [86]. Current research suggests that increased HuR contributes to diabetic microangiopathy through binding to VEGF and increasing its stability [18]. VEGF is a key regulator of angiogenesis [17]. Overactivation or overstabilization of VEGF leads to massive endothelial cell proliferation, which is the basis of pathological angiogenesis and consequently microangiopathy [87]. Amadio et al. showed that intraocular injection of lipoplexe (a nanosystem equipped with siRNA that inhibits HuR expression) in STZ-induced diabetic rats led to a significant decrease in retinal HuR and VEGF levels, achieving an improvement in diabetes-induced retinal symptoms [88]. Another study demonstrated that HuR and its binding protein Nox4 were significantly elevated in a model of high glucose-treated glomerular thylakoid cells and STZ-induced diabetic nephropathy. Further inhibition of HuR expression protects against hyperglycemia and renal function in a mouse model of type 1 diabetes mellitus [89]

#### 3.1.2 Tristetraprolin

Tristetraprolin (TTP), also known as zinc finger protein 36, is part of the TIS11 family [90]. The sequence of TTP, a 326 amino acid protein of the nucleocytoplasm, is characterized by the presence of two CCCH-type zinc finger structural domains [91]. It binds AU element-rich (AREs) mRNAs, targeting them for rapid mRNA decay in the cytoplasm [92]. TTP expression and/or activity is significantly reduced in advanced cancers [93] and diabetic microangiopathy [16]. The function of TTP has been reported to be the opposite of HuR. After conjugation to target mRNAs, HuR provides protection against their degradation, but TTP is a major player in disrupting the stability of mRNAs containing AU-rich elements (ARE) in the 3′ untranslated region [94,95]. Guo et al. demonstrated that the equilibrium between HuR and TTP was disrupted in the glomeruli of DKD patients and mice [16]. They found that in diabetic nephropathy, the TTP and HuR homeostasis affects podocyte injury and inflammation upon high-sugar exposure, possibly through the cleavage of IL-17 and apoptosis-associated proteins by increased caspase-3 [16,91,96].

#### 3.1.3 Heterogeneous ribonucleic acid proteins

Heterogeneous ribonucleic acid proteins (hnRNPs), including hnRNP A/B, hnRNP C and hnRNP G, are a group of RNA-binding proteins that assemble with newly generated transcripts within the nucleus of eukaryotic cells [97,98]. Their primary role is to facilitate the ripening of heterogeneous nuclear RNA (hnRNA/pre-mRNA) into messenger RNA (mRNA), stabilizing mRNA and controlling its translation during cytosolic transport [99]. Current research on hnRNPs focused mostly on cancer and neurodegenerative conditions such as Alzheimer's disease [100], but recently, studies suggest that the hnRNP family has a significant role in diabetic microangiopathy, without knowing its role [101]. According to Nutter et al., hnRNP K, hnRNP F and LIN28 are dysregulated in the DKD [32]. In contrast, other studies reported enhanced expression of hnRNP F, hnRNP K and angiotensin-converting enzyme-2 associated with insulin treatment [102]. In fact, up to now, the function of hnRNP has not been well understood, and one possible speculation regarding its mechanism in the pathogenesis of diabetic microangiopathy is that insulin inhibits the expression of renal Agt and the hypertrophy of renal proximal tubular cells caused by high glucose stimulation. Agt is serotonin and a precursor to angiotensin peptides that control blood pressure [103]. This repression is realized by a specific insulin response component in the promoter of Agt, where insulin-stimulated high expression of hnRNP F, hnRNP K specifically represses Agt transcription [33,104]. But in type 2 diabetic patients, this inhibition fails because of the existence of insulin resistance, leading to over-expression of Agt and consequent renal lesions such as albuminuria and tubular cell apoptosis [105]. However, further experimental evidence is still needed to support this view.

Collectively, these findings suggest that RBPs have a key function in the pathogenesis of diabetic microangiopathy (Table 1). It is generally clear that RBPs act through VEGF to affect angiogenesis, eventually leading to diabetic microvascular complications such as retinal microvascular proliferation and glomerular atherosclerosis. However, the key regulatory targets and mechanisms remain unknown. Further studies are needed to provide new insights.

**Table 1:** RBPs in diabetes complications.

RBPs	Expression	Target	Expression	Mechanism	Diseases	Reference
SRSF-6	↑	VEGF	↑	Alternative splicing	Diabetic retinopathy	[32,186]
HuR	↑	VEGF	↑	RNA stabilization、 mRNA translation	Diabetic retinopathy、	[141,187]
		GLUT1 mRNA				
hnRNP	↓	Bcl-x pre-mRNA、Sam68	–	RNA degradation, and stabilization、mRNA translation	Diabetic nephropathy	[188,189]
IGF2BP	↓	LAMB2、IGF	↓	RNA degradation, and stabilization、mRNA translation	Diabetic nephropathy	[156,190]
FTO	↑	DNA repair and cell survival-associated RNA	↓	RNA degradation	Diabetic nephropathy	[191,192]
TTP	↓	VEGF	↓	RNA degradation, and stabilization	Diabetic nephropathy, Diabetic retinopathy	[16]
eIF4E	↓	m7GpppN cap structure	–	mRNA translation	Diabetic retinopathy, Diabetic cardiovascular disease	[111]
CELF1	↑	Mis-spliced alternative splicing targets	–	Alternative splicing	Diabetic cardiovascular disease	[55]
LIN28	↓	let-7 miRNAs	↓	Alternative splicing 、mRNA translation	Diabetic cardiovascular disease	[193,194]
CUGBP1	↑	insulin receptor、 EMT-related genes	↑	mRNA translation、 Alternative splicing	Diabetic skeletal myopathy, Diabetic cardiomyopathy	[195]
RBFOX2	↑	dominant-negative	↑	Alternative splicing	Diabetic cardiomyopathy	[53]
MBNL-1	↑	calcineurin Aβ and SRF genes	↑	Alternative splicing	Diabetic wound healing	[170,196]
QKI	↑	–	–	mRNA stabilization and translation	Diabetic neuropathy	[126]
HuD	↓	GAP-43 mRNA	↓	post-transcriptional control of RNAs	Diabetic neuropathy	[173]
ZBP	↓	β-actin mRNA	↓	mRNA transport	Diabetic neuropathy	[197]
PTBP1	↑	certain pre-mRNAs	↑	Alternative splicing、 mRNA stabilization	Type 1 diabetes	[198]
IMP2	↓	PDX1 mRNAs	↓	RNA stabilization	Type 2 diabetes	[156]

RBPs: RNA binding proteins, SRSF-6: serine and arginine rich splicing factor 6, HuR: human antigen R, hnRNP: heterogeneous nuclear ribonucleoprotein, IGF2BP: IGF2 mRNA-binding protein, GLUT-1: glucose transporter 1, FTO: fat mass and obesity-associated protein, TTP: Tristetraprolin, eIF4E: eukaryotic translation initiation factor 4E, CELF1: CUGBP Elav-Like Family Member 1, CUGBP1: CUG-binding protein 1, RBFOX2: RBP Fox-1 homolog 2, MBNL-1: muscleblind like splicing regulator 1, QKI: Quaking, HuD: human antigen D, ZBP: Z-DNA-binding protein 1, PTBP1: polypyrimidine tract-binding protein 1, IMP2: IGF2 mRNA-binding protein.

**Table 2:** Potential Drugs Targeting RBPs and RBPs-RNA in Diabetes and Its Complications.

Drugs	Diseases	Function	Reference
CYC27	Diabetes	Modulate phosphorylation of RNA splicing-associated RBPs	[31]
Melatonin	Diabetes	Increase nuclear mammalian RBP HuD expression	[175]
Butyrate	Diabetic Nephropathy	Inhibit RBP P311 expression	[166]
Metformin	Diabetic Nephropathy	Promote the RBP MBNL1 expression	[179]
Sulfoximine quinazolines	Diabetes	Inhibit the phosphorylation of RBP eIF4E	[183]
Compound 49b	Diabetic Retinopathy	Increase RBP IGFBP-3 levels	[185]

RBPs: RNA binding proteins, HuD: human antigen D, MBNL1: muscleblind-like1, eIF4E: eukaryotic translation initiation factor 4E, IGFBP-3: insulin-like growth factor binding protein 3.

### 3.2 Macrovascular

Macrovascular disease, such as cardiovascular disease, is another major diabetic complication and a significant cause of death in people with diabetes [106]. Studies have shown that endothelial dysfunction is the earliest symptom of vascular injury and the key event in the pathogenesis of diabetes-related vascular disease [107]. Detailed mechanisms of macroangiopathy remain unexplored to date, however, recent research suggests that RBPs like LIN28A, EIF4E and CELF1 mediates endothelial dysfunction and macrovascular diseases through post-transcriptional RNA processing, especially alternative splicing [32].

#### 3.2.1 Eukaryotic initiation factor 4E

Eukaryotic initiation factor 4E (EIF-4E) is an mRNA cap-binding protein that is considered to be a universal promoter affecting mRNA-ribosome interactions and capture-dependent translation in eukaryotic cells [108]. By interacting with eukaryotic initiation factor 4G (EIF-4G), it specifically associates with the 7-methylguanosine cap located at the 5′ end of eukaryotic mRNA, thereby promoting the migration of mRNA to the ribosome and playing a core role in translation initiation [99]. Key regulatory roles for EIF-4E proteins, regulated from mTORC1 and EIF-4E binding proteins (EIF-4EBPs) [109]. Mammalian rapamycin complex 1 (mTORC1) is one of the key regulators of growth and metabolism in cells, and downstream effectors of its regulation include EIF-4EBPs and ribosomal protein S6K kinase (RP-S6K) [110]. Current research on EIF-4E focused on its role in cancer cell growth regulation and growth factors amplification [108]. Many works also support an important role of EIF-4E in diabetic macroangiopathy as well. Soliman et al. found that the mammalian rapamycin complex 1 (mTORC1) trophic sensing pathway is dysregulated in diabetes [110], while Seokwon Jo et al. demonstrated that the translation factor EIF-4G1 and EIF-4E protein damaged insulin secretion, glucose homeostasis and β-cell function in EIF-4G1 knockout [111]. This suggests the potential of EIF-4E and EIF-4G1 targeting therapies in reducing the risk of type 2.

#### 3.2.2 LIN28

LIN28 is a strongly conserved RBP involved for numerous biological procedures, for example, stem cell pluripotency [21,112,113]. Current research indicates that LIN28 can influence gene expression through RNA biogenesis, splicing and translation [114]. For example, LIN28 can bind to the circular RNA structure in pre-let7 RNA and block the production of adult let7 miRNA [113]. Meanwhile LIN28 can regulate alternative splicing by interacting with hnRNPA1 and hnRNPF as mentioned above [21]. Current research on LIN28 has focused on cancer, and Asghar Ali et al. demonstrated that optimization of LIN28A or LIN28B is the cause of the overall post-transcriptional down-regulation of the let-7 miRNA family observed in many cancers [21]. Apart from cancer, a new study suggests that LIN28 affects insulin resistance, glucose metabolism and mitochondrial function, contributing to protection from diabetes [115]. A possible hypothesis is that LIN28 could promote blood glucose uptake by tissue cells and reduce insulin resistance through affecting the processing and maturation of let-7 miRNAs [116,117]. Hwang, Y. J., et al. demonstrated that overexpression of LIN28a counteracted glucotoxicity-induced p-Akt and p-mTOR downregulation, thereby protecting islet cells in an in vitro model of the rat insulinoma cell line INS-1. LIN28 levels are reduced in diabetic cardiovascular lesions such as T1D mouse hearts [118], alongside apoptosis of cardiomyocytes, reduced contractile features, and increased mitochondrial cristae destruction. LIN28 upregulation caused changes in the Let-7b target gene and subsequently reversal of diabetic damages [116]. These findings provide evidence to support the protective effect of LIN28 in diabetic complications.

### 3.3 Other complications

In addition to the diabetic macrovascular and microangiopathy described above, RBPs are also involved with the development and course of almost all diabetic complications, for example, diabetic foot disease, neuropathy and cardiomyopathy. Diabetic foot is defined as an infection, ulcer or deep tissue destruction of the foot caused by neurological and vascular lesions in the lower limbs. It represents the one of the most serious acute diabetes complications and a major reason for hospitalization, death and disability in people with diabetes [[119], [120], [121]].

#### 3.3.1 Quaking

Quaking (QKI) is part of the signal transducer and activator of RNAs (STARs) family with a characteristic structural pattern containing a SH2 and a SH3 structural domain, an RNA binding pattern (e.g. KH structural domain) and sites of phosphorylation [122]. The functions of STARs include splicing of precursor mRNAs, mRNAs stabilization and translation. QKI is essential for sustaining function of the endothelial barrier as it increases in expression of β-catenin and VE-cadherinat in epithelial junctions, the process of which is disrupted in diabetic patients [105]. QKI has three main transcriptional isoforms, named QKI-5, QKI-6 and QKI-7 based on differences in the C-terminus. Generally, QKI- 5 is considered to be the most abundant [73]. QKI-5 is able to stabilize STAT3 mRNA by direct binding to the 3′UTR, thereby stabilizing VE-cadherin and activating VEGFR2, significantly promoting angiogenesis [123]. QKI-6, like QKI-5, has a positive effect on angiogenesis. It has been suggested that QKI-6 induces vascular smooth muscle cell formation by promoting HDAC7 splicing. Yang et al. showed that QKI-7 is highly expressed in coronary ECs from diabetic patients [124]. However, recent studies have found crucial function of QKI in the nervous system [125]. QKI is highly expressed in adult mouse glial cells, mainly astrocytes and oligodendrocytes [126,127], participating in the differentiation of myelin-forming oligodendrocytes and schwann cells through post-transcriptional regulation of gene expression, including mRNA splicing, stabilization, translation and stabilization [128]. Numerous studies support a strong link between QKI and central neuron system damage, and more intensive studies of it represent a promising strategy for the treatment of diabetic complications.

#### 3.3.2 RBP Fox-1 Homolog 2

RBP fox-1 homolog 2 (RBFOX2), along with RBFOX1, and RBFOX3, are members of the RBFOX family of RNA binding proteins [129]. RBFOX1 and RBFOX2 proteins are expressed in muscle, heart and brain tissues, acting as regulators of tissue-specific alternative splicing [130]. Current research has focused on the role of the RBFOX family in central nervous system pathologies. Bhalla K et al. found that mutations in the RBFOX1 gene are associated with mental retardation, epilepsy and autism, while RBFOX2 deletion leads to abnormal cerebellar development [32,131,132]. The latest studies noted that RBFOX2 is implicated in cardiac complications of diabetes: RBFOX2 regulates alternative splicing in diabetic heart disease by binding to the (U)GCAUG pattern in RNA [29]. Experiments by Wei et al. demonstrated that conditional deletion of RBFOX2 in mouse cardiomyocytes leads to dilated cardiomyopathy [133], but RBFOX2 is upregulated in the hearts of human patients with T2D. Nutter et al. found that RBFOX2 controls alternative splicing of genes with important roles in cardiac function and diabetic cardiomyopathy [53]. Also, a dominant-negative heterodimer blocking RBFOX2-mediated alternative splicing was generated in the diabetic heart, and its ectopic expression suppressed alternative splicing targeted by RBFOX2. Thus, one possible speculation regarding the mechanism of action of RBFOX2 in diabetic cardiovascular lesions is that by increasing the expression of the dominant-negative isoform to block RBFOX2-dependent alternative splicing results in the activity of RBFOX2 in diabetic cardiovascular lesions [53,134]. Low activity of RBFOX2 inhibits RBFOX2-dependent splicing and affects the processing of calcium ions by cardiomyocytes, ultimately leading to the development of diabetic cardiomyopathy [54]. However, the mechanism by which RBFOX2 causes diabetic cardiomyopathy is still not fully understood, and the relationship RBFOX2 and its dominant-negative levels is not yet elucidated. More studies are needed to support the speculation.

## 4 RNA-RBPs interaction in diabetes and its complications

### 4.1 In diabetes

**Figure 2:**
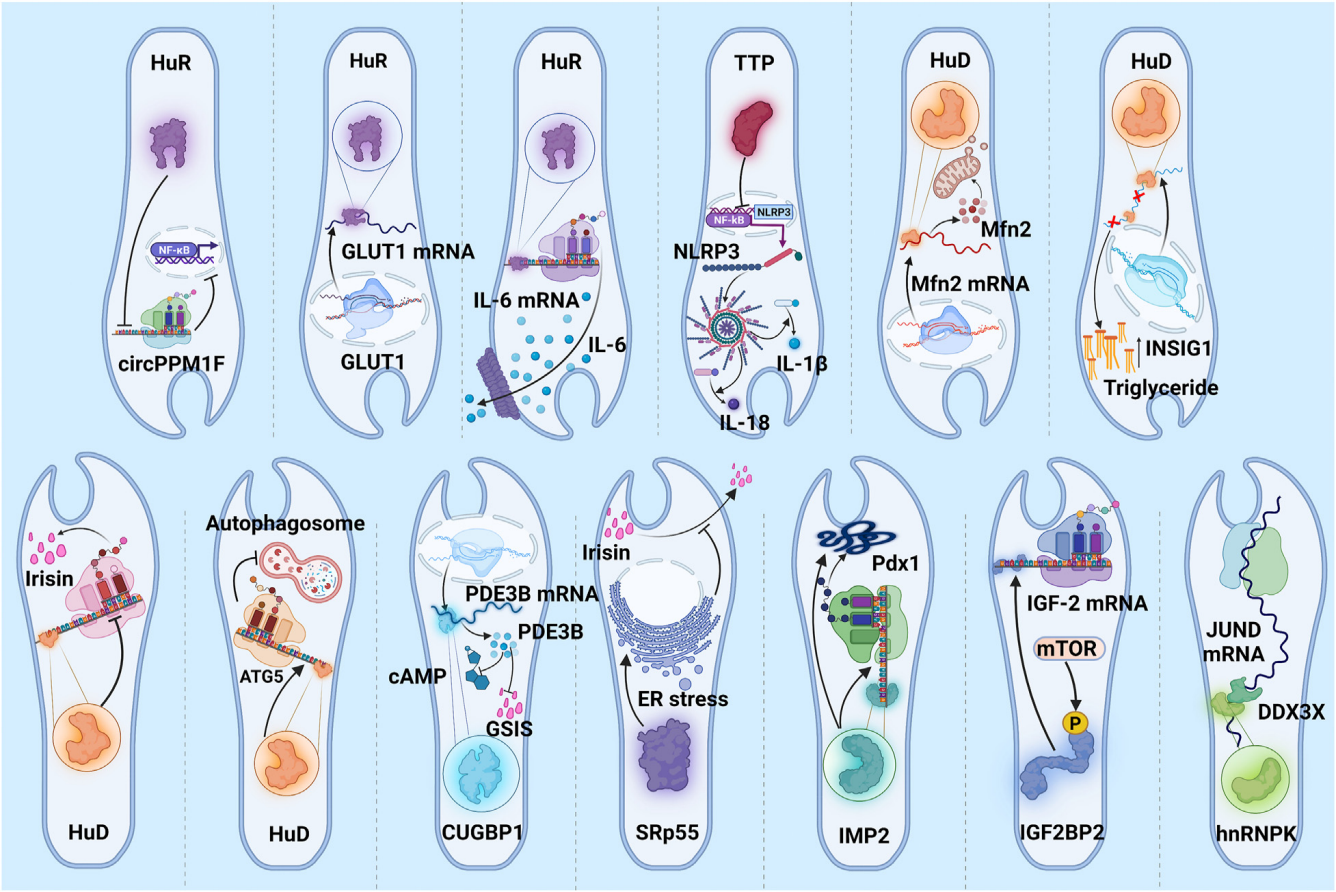
**RNA-RBPs Interaction in Diabetes.** Here, we summarized the main mechanisms on RNA-RBPs interaction in diabetes. HuR impairs circPPM1F translation, which promoted LPS-induced M1 macrophage activation via NF-κB signaling enhancement, thus exacerbating pancreas damage. Also, HuR interacts with the GLUT1 mRNA, potentially mediating post-transcriptional dysregulation and GLUT1-related metabolic disturbance in diabetes. Additionally, HuR might target IL-6 mRNA and enhances its stabilization, which promotes the development of inflammation in diabetes. TTP could inhibit NLRP3 expression in human macrophages by targeting the NLRP3 3′-untranslated region, which might influence the development of diabetes. Mfn2 play a critical role in diabetes through promoting glucose oxidation and insulin sensitivity. RBP HuD increased Mfn2 expression by binding to Mfn2 mRNA. Therefore, downregulation of HuD in diabetes contributes to impaired mitochondrial function, resulting in pancreatic β cell dysfunction. Also, downregulation of HuD accelerates the triglycerides production in pancreatic β cells by decreasing INSIG1 expression at the post-transcriptional level, thus enhancing nuclear translocation of the critical lipogenesis factor SREBP1c. In addition, HuD represses insulin translation through binding to the insulin mRNA and influences autophagosome formation in pancreatic β cells by promoting ATG5. CUGBP1 stabilizes the PDE3B mRNA, a phosphodiesterase that regulates cAMP hydrolysis, to reduce intracellular cAMP levels and impair GSIS. The depletion of SRp55 leads to β-cell apoptosis and impaired insulin secretion via regulating alternative splicing of multiple transcripts involved in insulin secretion and ER stress. IMP2 makes Pdx1 polypeptides stabilization via IGF2-AKT-GSK3β-PDX1 signaling pathway. mTOR phosphorylates IGF2BP2 and facilitates its binding to IGF-2 mRNA, thus enhancing IGF-2 expression. RBP hnRNPK targets the JUND mRNA in β cells with the bind of RNA helicase DDX3X, thus promoting β cell failure in T2D. RBPs: RNA binding proteins, HuR: human antigen R, circPPM1F: circular RNA protein phosphatase Mg2+/Mn2+ dependent 1F, LPS: lipopolysaccharide, NF-κB: nuclear factor kappa-B, GLUT1: glucose transporter 1, IL-6: interleukin 6, TTP: tristetraprolin, NLRP3: NLR family pyrin domain containing 3, Mfn2: Mitofusin-2, HuD: human antigen D, INSIG1: insulin-induced gene 1, SREBP1c: sterol regulatory element-binding protein 1, ATG5: autophagy-related gene 5, CUGBP1: CUG-binding protein 1, PDE3B: phosphodiesterase 3B, GSIS: glucose-stimulated insulin secretion, ER: endoplasmic reticulum, IMP2: IGF2 mRNA-binding protein 2, Pdx1: pancreatic and duodenal homeobox 1, mTOR: mammalian target of rapamycin, IGF2BP2: IGF2 mRNA-binding protein 2, IGF-2: insulin-like growth factor 2.

Diabetes is a group of metabolic diseases characterised by hyperglycaemia. Hyperglycaemia, in turn, is caused by a defect in insulin secretion or impairment of its biological action, or both [135,136]. Metabolic changes in diabetes offset the homeostasis in inflammation, ER stress, and oxidative stress, eventually activating pathogenic events such as fibrosis and EMT of cells [137]. Recently, in has been found that RNA-RBPs interactions get involved in the development of diabetes [32,49,138]. Here, we summarized several critical interaction in diabetes (Figure 2)

HuR is an important RBP in diabetes. A study in streptozocin-induced diabetic mice reported that HuR impairs circular RNA protein phosphatase Mg2+/Mn2+ dependent 1F (circPPM1F) translation, which promoted LPS-induced M1 macrophage activation via NF-κB signaling enhancement, thus exacerbating pancreas damage [139]. Glucose transporter-1 (GLUT-1) has critical function in diabetes for its ability to uptake glucose into cells [140]. Gantt, K.R., et al. found that HuR interacts with the glucose transporter 1 (GLUT1) mRNA, potentially mediating post-transcriptional dysregulation and GLUT1-related metabolic disturbance in diabetes [141]. IL-6 is a clear contributor to the diabetes progression, as multiple clinical trials identified increased IL-6 mRNA expression T2D patients [142,143]. HuR targets 3′-UTR region of IL-6 mRNA and enhances its stabilization, which promotes the development of inflammation in periodontitis [61]. However, the relationship between HuR and IL-6 in diabetes has not been evaluated. Whether HuR exerts a pro-inflammatory, pro-diabetic role through stabilization of IL-6 mRNA in diabetes is worth exploring. The NLRP3 inflammasome is an important regulator of inflammation, and its activation inducing many productions of pro-inflammatory molecular mediators such as IL-1β and IL-18, composing a key factor in diabetic inflammation [144,145]. A study identified that the RBP TTP could inhibit NLRP3 expression in human macrophages by targeting the AU-rich elements in the NLRP3 3′- UTR [146], which might influence the development of diabetes. Mitofusin-2 (Mfn2) play a critical role in diabetes through promoting glucose oxidation, insulin sensitivity, mitochondria and endoplasmic reticulum function [[147], [148], [149], [150]]. A study identified that RBP HuD increased Mfn2 expression by binding to 3′UTR of Mfn2 mRNA [138]. Downregulation of HuD in diabetes contributes to impaired mitochondrial function, resulting in pancreatic β cell dysfunction [138]. Downregulation of HuD also accelerates the triglycerides production in pancreatic β cells by decreasing insulin-induced gene 1 (INSIG1) expression at the post-transcriptional level, thus enhancing nuclear translocation of the critical lipogenesis factor sterol regulatory element-binding protein 1 (SREBP1c) [48]. Another study declared that HuD represses insulin translation through binding to the insulin mRNA 5′-UTR [151]. In addition, HuD influences autophagosome formation in pancreatic β cells by promoting autophagy-related gene 5 expression (ATG5) [152]. HuD might become one of the pivotal molecular regulators of autophagosome formation in pancreatic β cells.

Zhai K et al. found that RBP CUG-binding protein 1 (CUGBP1) level is increased in the db/db and high-fat diet mice models of diabetes [153]. CUGBP1 stabilizes the Phosphodiesterase 3B (PDE3B) mRNA, a phosphodiesterase that regulates cAMP hydrolysis, to reduce intracellular cAMP levels and impair glucose-stimulated insulin secretion (GSIS) [153]. Juan-Mateu, J., et al., found that the depletion of SRp55 leads to β-cell apoptosis and impaired insulin secretion via regulating alternative splicing of multiple transcripts involved in insulin secretion and ER stress such as BCL-2 [47]. RBP hnRNPK (phosphorylation in MEK/ERK signaling pathway) targets the JUND mRNA in β cells with the bind of RNA helicase DDX3X, thus promoting β cell failure in T2D [154].

Human insulin-like growth factor 2 (IGF2) mRNA binding protein family (IMPs/IGF2BPs) could influence RNA localization, stability, and translation, thus affecting T2D development [155]. A recent study declared that IMP2 directly binds to Pdx1 mRNA, thus enhancing its translation in an m6A dependent manner. Moreover, IMP2 makes Pdx1 polypeptides stabilization via IGF2-AKT-GSK3β-PDX1 signaling pathway [156]. A study showed that IGF2BP2 and IGF2 might have genetic effects in diabetes and diabetic nephropathy [157]. Another clinical trial suggested that the wild C allele of IGF2BP2 had a protective effect against T2DM in obese subjects of Chinese Han population [158]. IGF2BP2 can inhibit the expression of UCP1 by binding to UCP1 mRNA, thereby enhancing insulin resistant [159,160]. Furthermore, mTOR phosphorylates IGF2BP2 and facilitates its binding to IGF-2 mRNA, thus enhancing IGF-2 expression [160,161].

### 4.2 In diabetic complications

**Figure 3:**
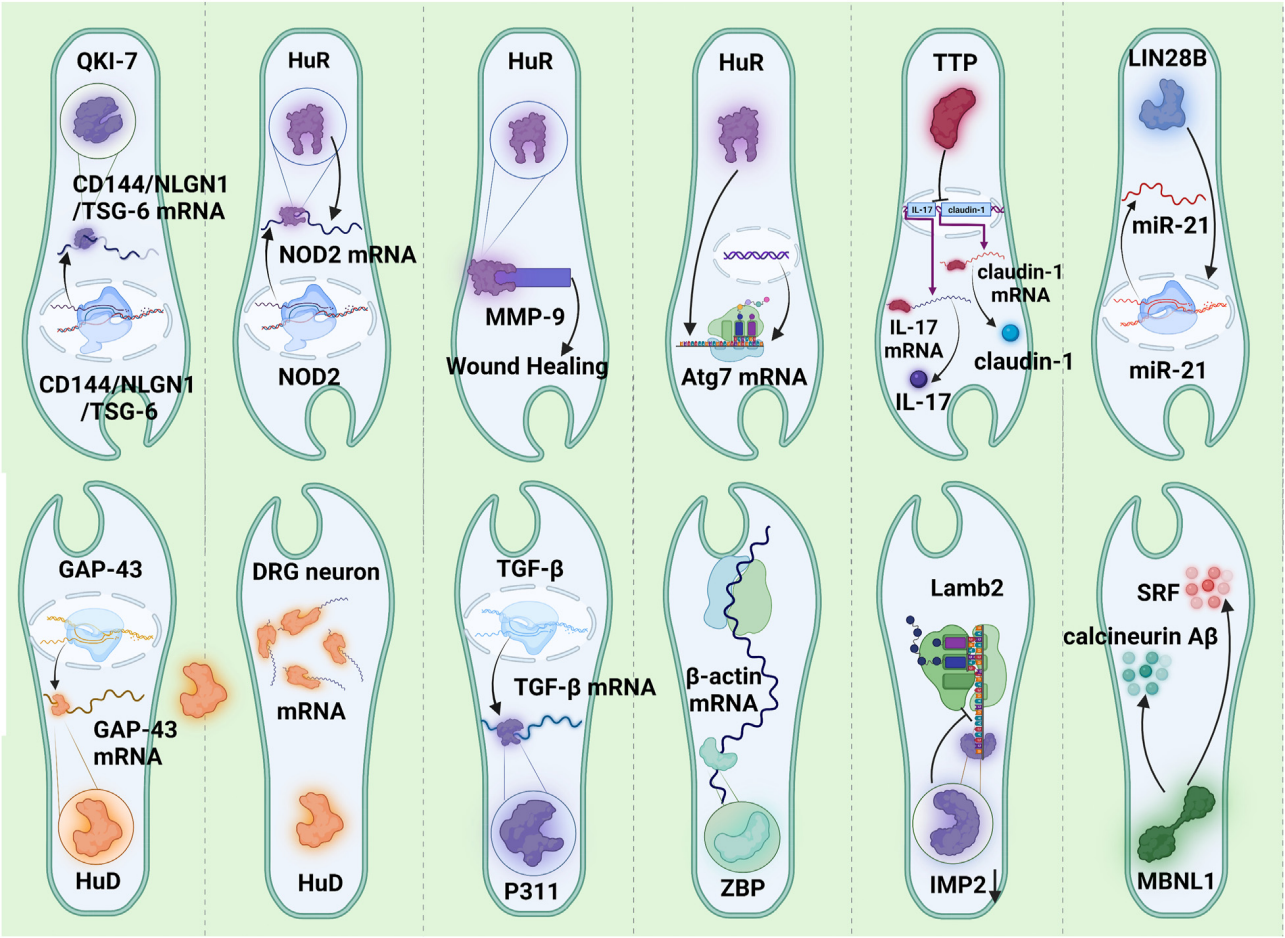
**RNA-RBPs Interaction in Diabetic Complications.** Here, we summarized the main mechanisms on RNA-RBPs interaction in diabetic complications. In diabetic endothelial dysfunction, upregulation of QKI-7 enhances mRNA degradation of CD144, NLGN1, and TSG-6. NOD2 is one of the critical signal molecules in DN. High glucose induces an increase in cytoplasmic HuR in rat glomerular mesangial cells in vitro. HuR could modulate renal injury to inflammation via binding to the 3′-UTR of NOD2, and the inhibition of HuR renders NOD2 mRNA unstable, reducing high glucose-induced NOD2 expression. In diabetic wound healing, HuR can bind to MMP-9, thus enhancing its stability, which promotes wound healing. In diabetic intervertebral disc degeneration, HuR was decreased in diabetic nucleus pulposus tissues and prompted Atg7 mRNA stability. Decreased TTP was demonstrated an important role in podocyte injury via binding to mRNAs (including IL-17 and claudin-1) influencing inflammation progression. LIN28B overexpression promotes the wound healing process by increasing VEGFA and miR-21. HuD downregulation makes GAP-43 mRNA decrease by post-transcriptional control of RNAs in diabetic neuropathy. HuD downregulation and HuB upregulation occurred in dorsal root ganglia sensory neurons, which may be associated with mRNA involved in DRG neuronal excitability. RBP P311 could promote the development of DN via stimulating TGF-β translation. RBP ZBP downregulation decreases β-actin mRNA by influencing mRNA transport in diabetic neuropathy. Reduced IMP2 level under conditions of high glucose significantly decreases Lamb2 mRNA translation in DN. RBP MBNL1 regulated calcineurin Aβ and SRF in myofibroblast differentiation, thus promoting wound healing. RBPs: RNA binding proteins, HuR: human antigen R, HuD: human antigen D, QKI-7: Quaking 7, TTP: Tristetraprolin, GAP-43: growth-associated protein 43, DRG: dorsal root ganglion, TGF-β: transforming growth factor-beta, Lamb2: laminin beta2, TSG-6: Tumor necrosis factor- (TNF) stimulated gene-6, NLGN1: Neuroligin1, NOD2: nucleotide-binding oligomerization domain 2, MMP-9: matrix metalloproteinase 9, Atg7: autophagy 7, IL-17: interleukin 17, IMP2: IGF2 mRNA binding protein 2, MBNL1: muscleblind-like 1, SRF: serum response factor.

There are some important RBPs-RNA interactions in diabetic complications (Figure 3). It is well known that Quaking-7 (QKI-7) upregulation is related to disrupted cell barrier, compromised angiogenesis and increased monocyte adhesion. A recent study discovered that the upregulation of QKI-7 enhances mRNA degradation of CD144, neuroligin 1 (NLGN1), and TNF-α-stimulated gene/protein 6 (TSG-6) in diabetes, altogether contributing to diabetic endothelial dysfunction [124]. Notably, knockdown of QKI-7 increases reperfusion following the induction of limb ischemia in diabetic mice, thus suggesting a promising therapeutic strategy for diabetic endotheliopathy [124,162].

Laminin-β2 (Lamb2) is a critical molecule for glomerular barrier permeability. Reduced IMP2 level under conditions of high glucose significantly decreases Lamb2 mRNA translation, which contributes to proteinuria in diabetic nephropathy (DN) [163]. NOD2 is one of the critical signal molecules in DN. A study found that high glucose induces an increase in cytoplasmic HuR in rat glomerular mesangial cells in vitro [164]. HuR could modulate renal injury to inflammation via binding to the 3′-UTR of NOD2, and the inhibition of HuR renders NOD2 mRNA unstable, reducing high glucose-induced NOD2 expression [164]. Constitutively active NADPH oxidase 4 (Nox4) is a major source of ROS that mediates hyperglycemia-induced mesangial cell (MC) fibrotic injury. HuR regulated DN progression via binding to AU-rich elements in Nox4 3′-UTR in kidneys from T1D animals [89]. Moreover, inhibition of HuR expression reduced MC injury and improved renal function [89]. The crucial function of HuR in DN has been proved in other studies [16]. Additionally, the decreased TTP was demonstrated the important role in podocyte injury via binding to mRNAs (including IL-17 and claudin-1) influencing inflammation progression in DN [16]. Additionally, a study found that HuR was decreased in diabetic nucleus pulposus tissues and prompted Atg7 mRNA stability via binding to the AU-rich elements in nucleus pulposus cells, which influenced diabetic intervertebral disc degeneration (DB-IVDD) in rats *in vivo* [165]. A study found that RBP P311 could promote the development of DN via stimulating TGF-β translation [166].

As already mentioned, HuR is particularly important for the development of diabetes and DN. A recent study evaluated its effects in diabetes on macrophage cellular/exosome-HuR [167]. They found that Exo-HuR could increase inflammatory and profibrogenic responses in fibroblast and cardiac fibrosis in mice. Under diabetic milieu, intravenous injection of Exo-HuR deficiency inhibited angiotensin II (ANG II)-induced cardiac inflammation and fibrosis [167]. Therefore, HuR might interact with ANG II to get involved in diabetes heart disease. In addition, RBP CELF1 was upregulated in the diabetic heart. Belanger, K., et al. identified extensive changes in alternative splicing patterns in T1D mouse hearts via utilizing genome wide approaches and many aberrantly spliced genes in T1D hearts have CELF1 binding sites, such as HDAC7 pre-mRNA [55].

In diabetic wound healing, LIN28 is a crucial RBP. It has been reported that LIN28B overexpression promotes the wound healing process by increasing VEGFA and miR-21 [168,169]. Additionally, Jennifer Davis et al. found that the expression of RBP muscleblind-like1 (MBNL1) was significantly increased in the area of injured skin in the mouse skin injury model during wound healing [170]. Mechanistically, they found that MBNL1 regulated calcineurin Aβ and serum response factor (SRF) in myofibroblast differentiation, thus promoting wound healing [170]. In addition, a study investigated the relationship between RBP HuR and MMP-9 in diabetic wound healing [171]. It is well known that MMP-9 plays a crucial role in the degradation of ECM components and growth factors [172]. They found that HuR can bind to MMP-9, thus enhancing its stability, which promotes wound healing [171]. In diabetic neuropathy, a study demonstrated that, in diabetes, HuD downregulation and HuB upregulation occurred in dorsal root ganglia sensory neurons, which may be associated with mRNA involved in DRG neuronal excitability [173].

## 5 Potential drugs targeting RBPs and RBPs-RNA in diabetes and its complications

Considering the critical role of RBPs and the interaction between RBPs-RNA in the progression of T1D and T2D diabetes, as well as in diabetic complications, drug development based on RBP regulatory pathways attracted much attention. In this part, we will discuss several RBPs and RBPs-RNA based drugs under investigation for treatment of diabetes and its complications (Table 2).

It has been reported that CYC27 is a synthetic derivative of marine bromophenol isolated from red alga *Rhodomela confervoides*. A study found that CYC27 exerted anti-diabetic effects by sensitizing insulin signaling pathways and regulating alternative splicing associated with RBPs [31]. Analysis of protein–protein interactions network based on STRING database and shown Cytoscape revealed that CYC27 modulates alternative splicing via phosphorylating RBPs, including upregulated phosphorylation of Cstf3, Srrt, Fip1l1, Srsf5, Srrm2, Srsf2 and Rbm25, and the downregulated phosphorylation of Prpf38a, Ddx23, Prpf3 and Hnrnph1. CYC27 mediated RBP phosphorylation may be the central mechanism of its anti-diabetic effect. Melatonin participates in the maintenance of glucose homeostasis to slow the progression of T2D [174]. A study found that melatonin increases nuclear mammalian HuD expression, thus mediating insulin synthesis in rat insulinoma INS-1E cells [175]. Based on these results, melatonin may be a potential anti-diabetic RBP modulator. RBP P311 stimulates translation of transforming growth factor-β1 (TGF-β1), a critical driver of the profibrotic signaling cascades [166]. The study found that butyrate alleviates DN by inhibiting P311 and TGF-β1 in the kidney of db/db mice as well as high glucose-induced SV40-MES-13 cells [166]. Metformin is a biguanide derivative and the current first line choice for treatment of T2D [176,177]. The extremely serious complication after taking metformin is lactic acidosis [178]. A study found that RBP MBNL1 can bind to miR-130a-3p, thus increasing its stability [179]. MiR-130a-3p decreases signal transmission and transcription activation factor 3 expression in renal tubular epithelial cells, which plays a crucial role in the pathogenesis of DN [180,181]. Recently, Jiang, X., et al. found that metformin can promote the RBP MBNL1 expression to reduce the senescence of renal tubular epithelial cells in DN [179]. It has been reported that eIF4E is a RBP responsible for initiating translation [182]. The phosphorylation of eIF4E is the key event which initiates translation. Mnk1 and Mnk2 are protein kinases responsible for phosphorylating eIF4E. A study found that sulfoximine quinazolines could inhibit Mnk1 and Mnk2 activity, thus modulating eIF4E function in the treatment of metabolic diseases including diabetes [183]. Compound 49b is a novel PKA-activating drug. In the oxygen-induced retinopathy of prematurity model, insulin-like growth factor binding protein 3 (IGFBP-3) has antiapoptotic function on retinal endothelial cells [184]. Study found that compound 49b could increase IGFBP-3 levels thereby preventing diabetes-induced apoptosis [185]. In short, compound 49b might be an important therapeutic drug in diabetic retinopathy.

In conclusion, RBPs play a vital role in diabetes and its complications. The function of RBPs provides a promising therapeutic strategy to treat diabetes and its complications. Emerging therapies targeting RBPs and RBP-RNA interactions are under lab and clinical evaluations.

## 6 Conclusion

In this review, we summarize the important RBPs involved in diabetes and diabetic complications, and we discuss the RBPs-RNA interaction in diabetes. At the same time, several drugs relevant to RBPs and RBPs-RNA in diabetes therapy are also illustrated. Recent studies demonstrate that RBPs play a vital role in the development of diabetes and diabetic complications. With the development of biological and molecular science, an increasing number of RBPs and RNA (targets of RBPs) in diabetic conditions are discovered, giving us a better understanding of the occurrence of diabetes and its complications. Meanwhile, RBP-based therapies in diabetic patients are under investigation. Notably, drugs targeting the same RBP can have tissue-specific effects, reflecting the multi-functional nature of RBPs. The mechanism of function and regulatory networks of RBPs deserves further investigation.

At present, drug or genetic intervention strategies targeting RBPs are still in the infancy, especially in treatment of diabetes and diabetic complications. RBPs affect the post-transcriptional degradation of RNA and the expression of its downstream genes, thereby inhibiting the occurrence and development of diabetes mellitus and complications, but the specific mechanisms still need to be further studied. In particular, the different alternative splicing regulation of RBPs leads to the complexity of its protein function, and more research is needed to elucidate the role and mechanism of RBPs and RNA-RBPs in diabetes mellitus, diabetic complications, and other metabolic diseases. In summary, posttranscriptional modulation by RBPs is emerging as a vital mechanism in the pathogenesis of diabetes and its complications. The intricate web of regulation has provided a promising option as a therapeutic target for diabetic patients.

## Author contributions

Conceptualization, J.Z. and P.Y.; writing—original draft preparation, X.C., J.W., and Z.L.; adapt the text and figures, Z.L., J.H., P.X., Y.S., and X.L.; writing—review and editing, P.Y.; project administration, P.Y.; funding acquisition, J.Z. and P.Y. All authors have read and agreed to the published version of the manuscript.

## Funding

This work was supported by the Natural Science Foundation in Jiangxi Province grant [grant numbers No.202002BAB216022 to J.Z., No.20192ACBL21037 and No.202004BCJL23049 to P.Y.]; the National Natural Science Foundation of China [grant number No.82160371 to J.Z., No.82100869 to P.Y., and No.21866019 to J.Y.M].
